# Application of a chemical probe to detect neutrophil elastase activation during inflammatory bowel disease

**DOI:** 10.1038/s41598-019-49840-4

**Published:** 2019-09-16

**Authors:** Bethany M. Anderson, Daniel P. Poole, Luigi Aurelio, Garrett Z. Ng, Markus Fleischmann, Paulina Kasperkiewicz, Celine Morissette, Marcin Drag, Ian R. van Driel, Brian L. Schmidt, Stephen J. Vanner, Nigel W. Bunnett, Laura E. Edgington-Mitchell

**Affiliations:** 1 Department of Biochemistry and Molecular Biology, Bio21 Molecular Science and Biotechnology Institute, The University of Melbourne, Parkville, VIC Australia; 2 Drug Discovery Biology, Monash Institute of Pharmaceutical Sciences, Monash University, Parkville, VIC Australia; 3 Medicinal Chemistry, Monash Institute of Pharmaceutical Sciences, Monash University, Parkville, VIC Australia; 4 Department of Cellular Immunology, Institute of Experimental Immunology, University Hospital Bonn, Bonn, Germany; 5 Department of Bioorganic Chemistry, Wroclaw University of Technology, Wroclaw, Poland; 6 Gastrointestinal Diseases Research Unit, Kingston General Hospital, Queen’s University, Kingston, ON Canada; 7 Department of Oral and Maxillofacial Surgery, New York University College of Dentistry, Bluestone Center for Clinical Research, New York, New York, USA; 8 Departments of Surgery and Pharmacology, Columbia University, New York, New York, USA; 9 Department of Pharmacology and Experimental Therapeutics, The University of Melbourne, Parkville, VIC Australia

**Keywords:** Proteases, Chronic inflammation, Diagnostic markers, Ulcerative colitis

## Abstract

Neutrophil elastase is a serine protease that has been implicated in the pathogenesis of inflammatory bowel disease. Due to post-translational control of its activation and high expression of its inhibitors in the gut, measurements of total expression poorly reflect the pool of active, functional neutrophil elastase. Fluorogenic substrate probes have been used to measure neutrophil elastase activity, though these tools lack specificity and traceability. PK105 is a recently described fluorescent activity-based probe, which binds to neutrophil elastase in an activity-dependent manner. The irreversible nature of this probe allows for accurate identification of its targets in complex protein mixtures. We describe the reactivity profile of PK105b, a new analogue of PK105, against recombinant serine proteases and in tissue extracts from healthy mice and from models of inflammation induced by oral cancer and *Legionella pneumophila* infection. We apply PK105b to measure neutrophil elastase activation in an acute model of experimental colitis. Neutrophil elastase activity is detected in inflamed, but not healthy, colons. We corroborate this finding in mucosal biopsies from patients with ulcerative colitis. Thus, PK105b facilitates detection of neutrophil elastase activity in tissue lysates, and we have applied it to demonstrate that this protease is unequivocally activated during colitis.

## Introduction

Neutrophil elastase (NE) is a serine protease found within azurophilic granules of neutrophils^1^. During infection, active NE contributes to killing of pathogens, and mice lacking NE are more susceptible to bacterial and fungal infections^1^. NE also mediates inflammation by processing cytokines, chemokines and growth factors^1^. Furthermore, NE cleaves the extracellular N termini of protease-activated receptors (PARs), a family of G protein-coupled receptors (GPCRs), to initiate cellular signaling events that lead to inflammation and pain^2–4^.

NE has recently been implicated in the pathogenesis of inflammatory bowel diseases (IBD), which are characterized by chronic and relapsing inflammation in the gastrointestinal tract^5^. IBD comprises ulcerative colitis (UC) and Crohn’s disease (CD), both of which are associated with diarrhea, rectal bleeding, increased urgency and pain. Mice lacking one copy of NE and a closely related neutrophil serine protease, proteinase 3 (PR3), exhibit improved symptoms in mouse models of colitis^6^. Enforced expression of elafin, an endogenous serine protease inhibitor, either by intracolonic administration of adenoviral vectors or introduction of elafin-expressing lactic acid bacteria, resulted in attenuation of symptoms in mouse models of colitis^6^. Treatment with a NE-selective inhibitor also reduced colitis symptoms^7^.

Colonic mucosal biopsies from patients with IBD exhibit elevated NE expression compared to healthy controls at both mRNA and protein levels^8,9^. Because NE is expressed as an inactive zymogen and can be tightly controlled by endogenous inhibitors once activated, measures of mRNA or total protein expression rarely reflect the pool of active functional enzyme^10^. Thus, tools to measure the specific activity of NE are required.

Commercially available chromogenic and fluorogenic substrate probes, including AAPV-p-nitroanilide and BODIPY-FL-elastin, respectively, have indicated an increase in elastase-like activity in biopsies from UC and CD patients and in mouse models of IBD^6,7,11,12^. However, these probes lack specificity and can be cleaved by multiple proteases^5,10^. Because the probes do not bind directly to the proteases upon cleavage, it is difficult to estimate the proportion of cleavage that is dependent on NE. Thus, improved tools are required to specifically measure NE activation and to improve our understanding of its functions during IBD pathogenesis.

**Figure 1:**
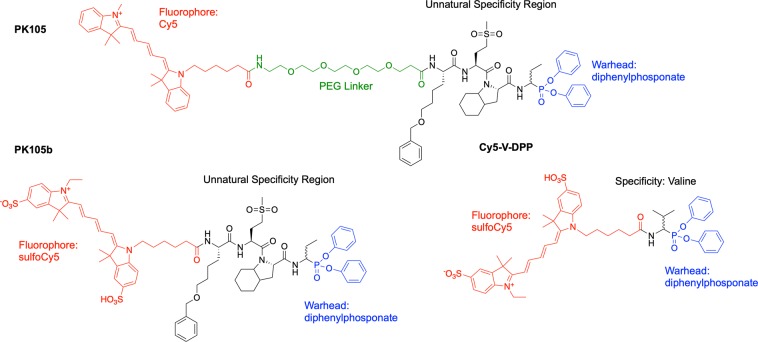
Structures of PK105^16^, PK105b, and Cy5-V-DPP^13^.

We previously applied a fluorescent activity-based probe (ABP) for NE, Cy5-V-DPP, to track NE activation during colitis^13^. This probe contained a sulfonated cyanine 5 (Cy5) fluorophore and a P1 valine residue coupled to a diphenylphosphonate electrophile (DPP; ‘warhead’) that reacts with the active site serine of active NE in a covalent, irreversible manner (Fig. 1). While Cy5-V-DPP efficiently labeled recombinant NE and NE in cells with high expression (*e.g*., bone marrow), we had little success in detecting NE activity in colitis tissues, presumably due to a lack of sensitivity. Use of hybrid combinatorial substrate libraries allowed for optimization of the NE substrate recognition sequence, which was coupled to Cy5 and the DPP warhead to generate the ABP, PK105^14–17^. While PK105 has been extensively characterized in purified neutrophils, its efficacy and specificity for NE in more complex tissue lysates has not been explored until now.

Herein, we characterized the reactivity profile of PK105b, an analogue of PK105, and explored its efficacy in complex tissue lysates. We applied PK105b in a mouse model of acute colitis and in human mucosal biopsies from patients with IBD to clearly measure activation of NE in inflamed tissue.

## Results

### Selectivity and potency of PK105b against purified serine proteases

We synthesized an analogue of our previously published PK105 probe^16^, referred to as PK105b, in which the polyethylene glycol (PEG) linker was omitted and sulfoCy5 was used in place of the unsulfonated version (Fig. 1). This change allowed for a more direct comparison to our Cy5-V-DPP probe^13^, which included sulfoCy5 and no PEG. The only difference between the two probes compared in this study is the specificity region: Cy5-V-DPP contains a single P1 valine residue, while PK105b contains a tetrapeptide consisting of non-natural amino acids (Nle(OBzl)-Met(O)2-Oic-Abu) (Fig. 1).

**Figure 2:**
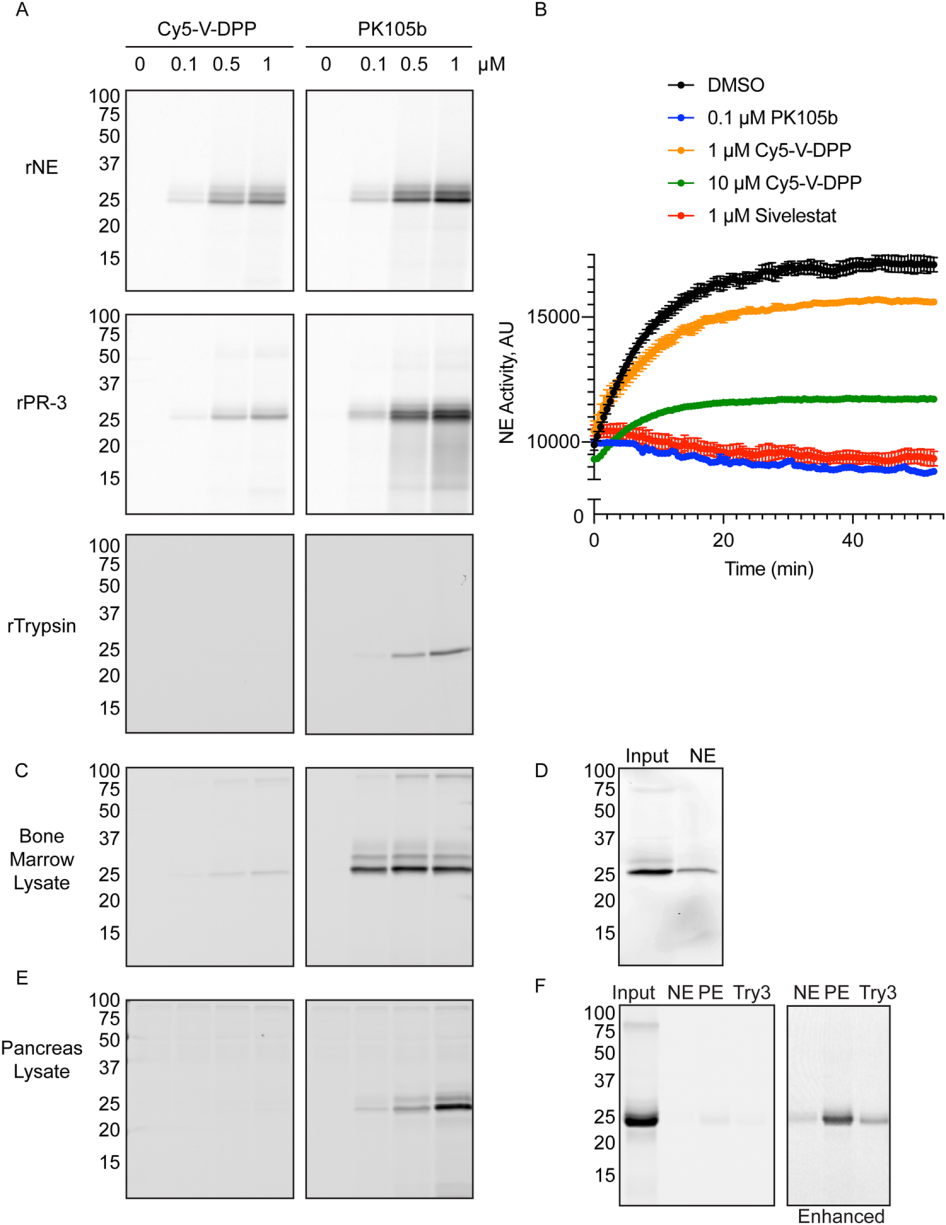
Comparison of selectivity profile for PK105b and Cy5-V-DPP. (**A**) Recombinant serine proteases (neutrophil elastase, proteinase 3, or trypsin) were incubated with increasing concentrations of Cy5-V-DPP or PK105b and binding was assessed by in-gel fluorescence. (**B**) Kinetic measurements of recombinant neutrophil elastase activity using the fluorogenic substrate probe, AAPV-AMC in the presence of DMSO (vehicle control) or the indicated concentration of PK105b, Cy5-V-DPP or Sivelestat. Error bars are shown as mean ± SEM. (**C**,**E**) Lysates from mouse bone marrow or pancreas were incubated with increasing concentrations of Cy5-V-DPP or PK105b and binding was assessed by in-gel fluorescence. (**D**,**F**) PK105b-labeled lysates were immunoprecipitated with antibodies for neutrophil elastase, pancreatic elastase (PE) or trypsin 3 (Try3), as indicated. In (**F**), gain settings for pulldowns were enhanced in order to observe faint bands (right panel). Note: Cy5-V-DPP and PK105b labeling are depicted at equal gain settings, set independently for each sample type.

We tested the reactivity of the PK105b against recombinant serine proteases, and compared its potency with Cy5-V-DPP. After incubation of proteases with increasing concentrations of probes for 30 minutes, the proteins were resolved by SDS-PAGE and binding was detected by in-gel fluorescence. Both probes clearly labeled NE and PR-3 in a concentration-dependent manner (Fig. 2A), though PK105b was more potent than Cy5-V-DPP. PK105b also clearly labeled trypsin, another serine protease, while trypsin binding by Cy5-V-DPP was negligible (Fig. 2A).

Using a commercially available fluorogenic substrate probe, AAPV-AMC, we compared the ability of the two probes to inhibit recombinant NE. Like Sivelestat, a commonly used NE inhibitor, PK105b immediately inhibited rNE activity at 0.1 µM while Cy5-V-DPP only partially inhibited rNE activity at 1 and 10 µM (Fig. 2B). These results indicate that PK105b binds to rNE more rapidly and more potently than Cy5-V-DPP. As Cy5-V-DPP and PK105b have identical DPP warheads, linkers, and sulfoCy5 fluorophores, we can attribute the improved potency and kinetics of PK105b to its extended non-natural specificity region, which likely fits better within the NE active site than valine alone.

### Selectivity and potency of PK105b in normal tissue lysates

We compared the ability of PK105b and Cy5-V-DPP to detect protease activity in lysates prepared from mouse bone marrow. Two proteases were labeled by PK105b (Fig. 2C), and the 25-kDa protein was confirmed to be NE by immunoprecipitation with an NE-specific antibody (Fig. 2D). NE was specifically labeled by Cy5-V-DPP, but with a much lower potency than PK105b. We also examined the reactivity of the probes in lysates prepared from mouse pancreas, a tissue rich in serine proteases. With PK105b, we observed strong labeling of 25-kDa proteins that was much more apparent than labeling by Cy5-V-DPP (Fig. 2E). Immunoprecipitation of PK105b-labeled lysates revealed that the target proteases consisted of a combination of NE, pancreatic elastase (PE), and trypsin 3 (Try3, also known as PRSS3 or mesotrypsin; Fig. 2F). Thus, PK105b exhibits a dramatic improvement in labeling efficiency in protein lysates over Cy5-V-DPP. In addition to the improved binding kinetics demonstrated above, the improved labeling profile of PK105b may also be due in part to enhanced stability of the compound afforded by the non-natural specificity region, which may be less prone to degradation than Cy5-V-DPP.

### Validation of PK105b in inflamed tissues

**Figure 3:**
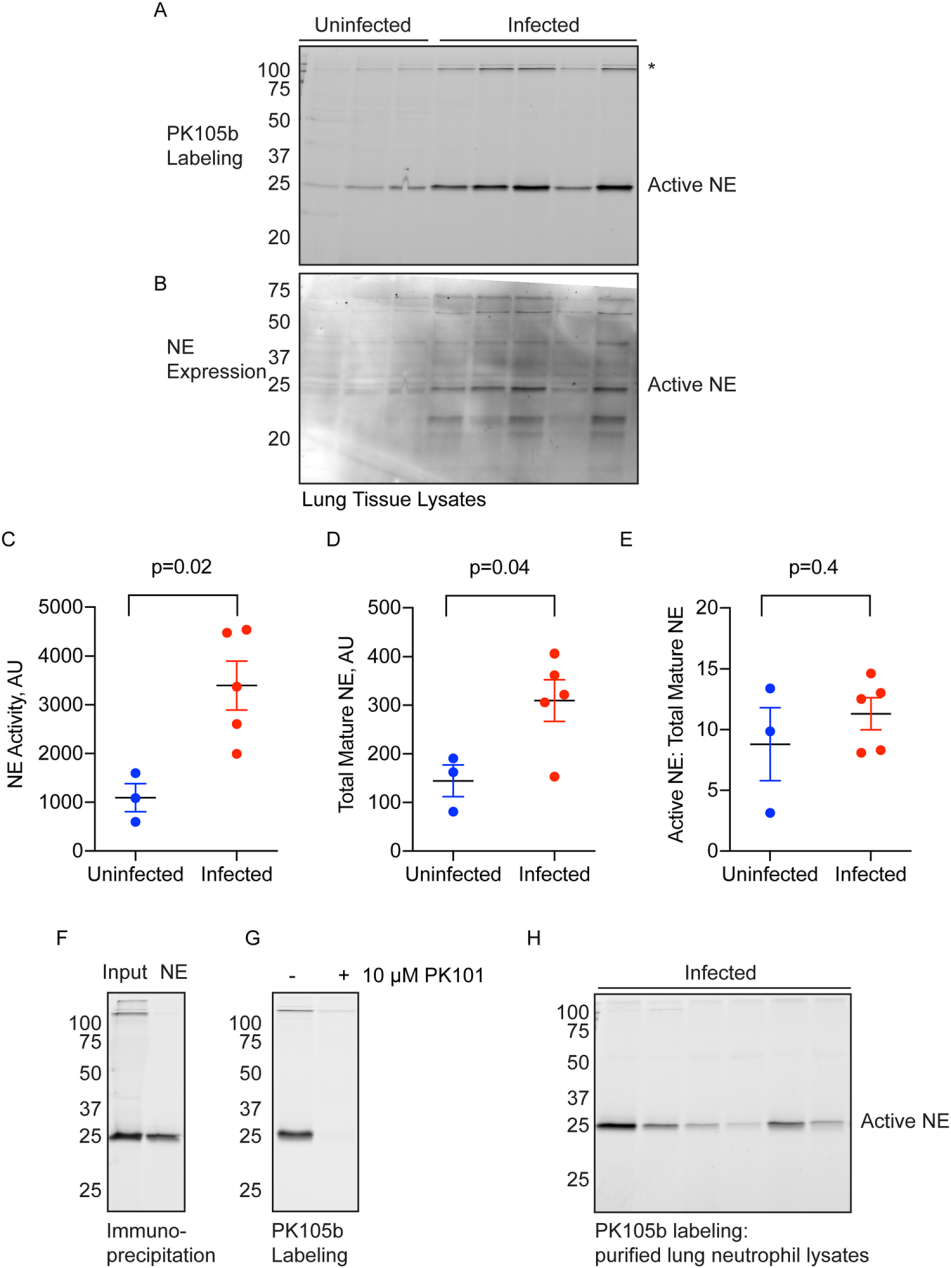
Detection of NE activity in *L. pneumophila*-infected lung tissue. (**A**) Lysates prepared from uninfected or *L. pneumophila*-infected lungs were labeled with 1 µM PK105b and analyzed by in-gel fluorescence (n = 3–5). *Indicates high-molecular weight species of unknown identity. (**B**) Total NE expression in the samples in (**A**) was measured by immunoblotting with an NE-specific antibody. (**C**,**D**) Densitometry analysis of active NE in (**A**) and total mature NE in (**B**), respectively. (**E**) Ratios of active to total mature NE in the samples. (**F**) Labeling of NE by PK105b in infected lung tissue was confirmed by immunoprecipitation with an NE-specific antibody. (**G**) Competetion of PK105b labeling in an infected lung lysate with PK101. (**H**) PK105b labeling in neutrophils isolated from infected lung tissue, as analyzed by in-gel fluorescence (refer to Fig. S1 for sorting strategy). Error bars are shown as mean ± SEM.

To determine the effectiveness of PK105b to measure NE activation in inflamed tissues, we used a mouse model of *Legionella pneumophila* infection. *L. pneumophila* infection may result in Legionnaire’s disease, a common cause of community or hospital-acquired pneumonia, and is associated with high neutrophil infiltration in the lung^18,19^. PK105b labeling was significantly increased in lysates prepared from infected lung tissues compared to uninfected lungs (Fig. 3A,C). The identity of the major 25-kDa species was confirmed to be NE by immunoblotting (Fig. 3B,D,E) and immunoprecipitation (Fig. 3F) with an NE-specific antibody. We also confirmed that PK105b binding was mediated by the DPP warhead and the specificity region, as the labelling could be competed by pre-treatment with PK101^14,15^, a biotinylated (non-fluorescent) analogue of PK105 (Fig. 3G). As neutrophils are the predominant source of NE during *L. pneumophila* infection, we also examined PK105b labelling in neutrophils from infected-lungs, which were sorted by flow cytometry at >97% purity (Fig. S1). Within this population of cells, we observed specific labeling of NE by PK105b (Fig. 3H).

**Figure 4:**
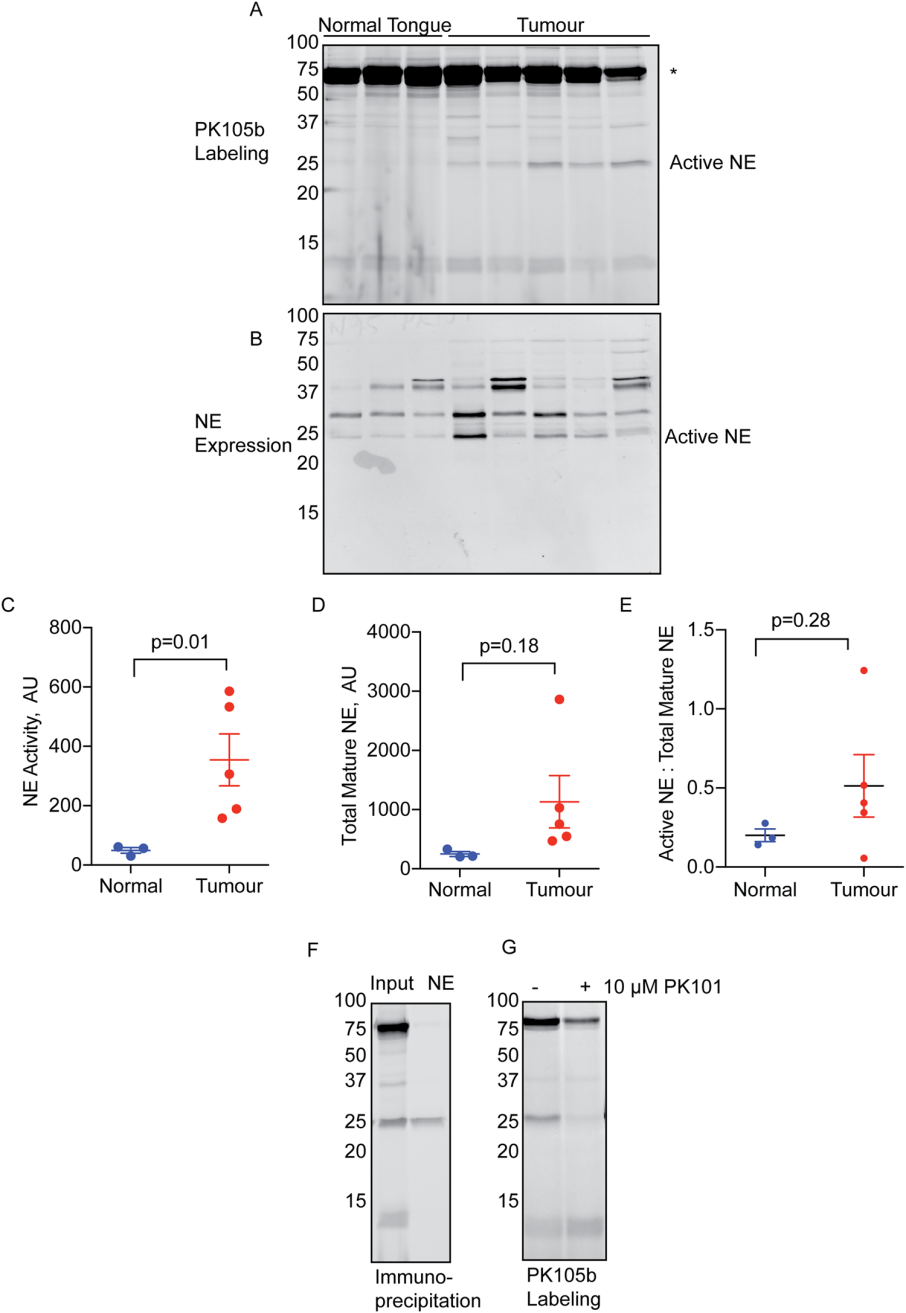
Detection of NE activity in oral cancer tissues. (**A**) Lysates prepared from normal mouse tongues or HSC-3 oral cancer tissues were labeled with 1 µM PK105b and analyzed by in-gel fluorescence (n = 3–5). *Indicates high-molecular weight species of unknown identity. (**B**) Total NE expression in the samples in (**A**) was measured by immunoblotting with an NE-specific antibody. (**C**,**D**) Densitometry analysis of active NE in (**A**) and total mature NE in (**B**), respectively. (**E**) Ratio of active to total mature NE in the samples. Error bars are shown as mean ± SEM. (**F**) NE labelling in cancer tissue was confirmed by immunoprecipitation with an NE-specific antibody. (**G**) Competition of PK105b labeling in oral cancer tissue lysate with PK101.

We next determined the utility of PK105b to detect NE activation in a cancer setting, which is also rich in neutrophils^20–22^. Specifically, we utilized a mouse orthotopic xenograft model of oral squamous cell carcinoma in which human cancer cells (HSC-3) were injected into the tongue^23^. In this context, we observed clear labeling of a 25-KDa species in tumor tissues, but not normal tongue tissues (Fig. 4A,C). This species coincided with the size of mature NE as determined by immunoblot (Fig. 4B,D,E) and immunoprecipitation (Fig. 4F) with an NE-specific antibody. Several other high-molecular weight species were abundantly labeled by PK105b in these lysates (Fig. 4A), but they have not yet been identified. Nonetheless, they are likely to be binding to PK105b through the DPP and specificity region, and not Cy5, as binding could largely be competed with PK101, the non-fluorescent PK105 analogue (Fig. 4G).

### Application of PK105b to measure NE activation in experimental colitis

**Figure 5:**
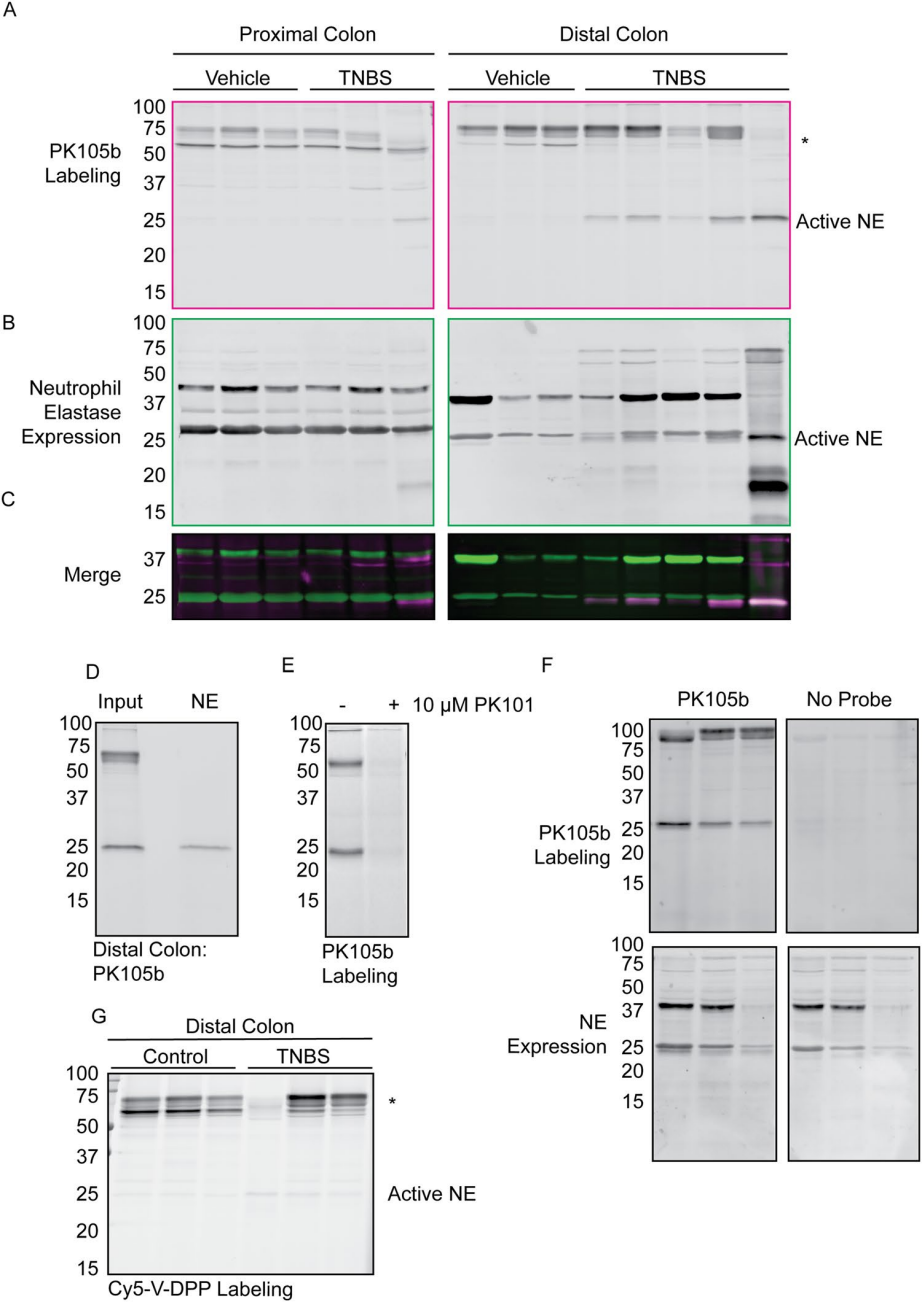
Application of PK105b to measure NE activation in acute experimental colitis. (**A**) Distal or proximal colons were excised from mice with acute colitis induced by TNBS and labeled *ex vivo* with 1 µM PK105b. Probe binding was detected by in-gel fluorescence (n = 3–5). *Indicates high-molecular weight species of unknown identity. (**B**) Immunoblotting of samples in (**A**) with an NE-specific antibody to reveal total NE expression. (**C**) Merged image of PK105b labeling (active NE; magenta) and immunoblot (total NE expression; green). (**D**) Immunoprecipitation of PK105b-labeled inflamed distal colon lysate with a NE-specific antibody. (**E**) Competition of PK105b labeling in inflamed distal colon lysates with PK101. (**F**) In-gel fluorescence and NE immunoblot of distal colon lysates with or without PK105b labeling. (**G**) Distal colon lysates from A labeled with Cy5-V-DPP.

Having validated PK105b in inflamed mouse tissues, we next applied this probe to investigate NE activation during acute experimental colitis induced by trinitrobenzenesulfonate (TNBS). As expected, mice exhibited loose stools, delayed defecation, weight loss (Fig. S2A), and colon shortening (Fig. S2B). We also observed damage to the mucosa by histological evaluation, as well as edema and inflammatory infiltrate (Fig. S2C). We analyzed colon lysates for NE activation by PK105b labeling and measurement of in-gel fluorescence. In proximal colons from healthy and inflamed mice, we observed little PK105b labeling. By contrast, in the distal region of inflamed colons, which is most affected in the TNBS model, we observed clear labeling of a 25-kDa protein (Fig. 5A). This band was virtually absent in distal colons of healthy mice that received vehicle instead of TNBS (Fig. 5A). We confirmed the identity of the protease to be NE by immunoprecipitation with an NE-specific antibody (Fig. 5D). All of the PK105b bands could be competed by PK101, the non-fluorescent PK105 analogue (Fig. 5E).

We also transferred the fluorescent gels to nitrocellulose membranes in order to immunoblot the samples for total NE expression. In all proximal colons and in healthy distal colons, we observed bands at 37 and 25 kDa (Fig. 5B). In the TNBS-treated distal colons, a new band appeared just below the 25-kDa protein. Only the lower species was labeled by PK105b, as revealed by overlay of the Cy5 fluorescence (Fig. 5C) and immunoprecipitation (Fig. 5D). To verify that the appearance of this smaller NE species was not an artefact of probe labeling, we immunoblotted inflamed distal colon samples in the presence and absence of PK105b. The smaller species was detected regardless of the presence of PK105b (Fig. 5F). Taken together, these data suggest that NE is subject to trimming in inflamed regions of the colon that permits its activation and thus its reaction with the PK105b probe.

**Figure 6:**
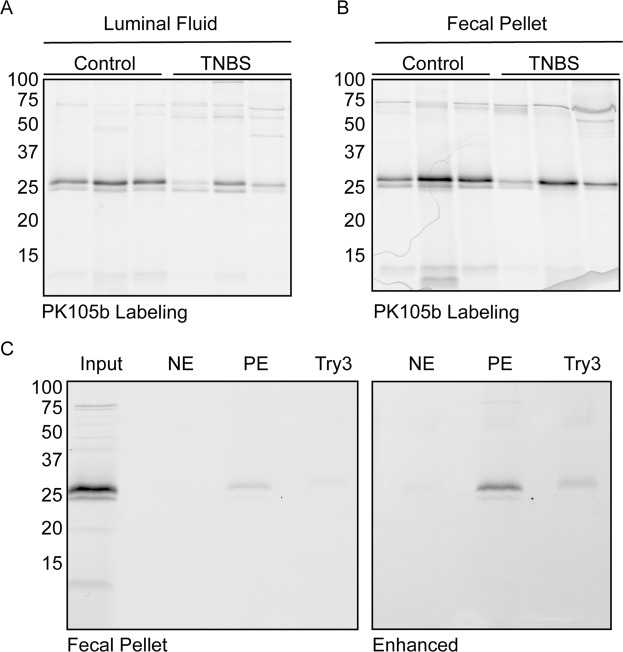
Luminal protease activity in acute experimental colitis. (**A**,**B**) Labeling of luminal fluids or fecal pellets from control or TNBS-treated mice with PK105b. (**C**) Immunoprecipitation of PK105b-labeled fecal samples with NE, PE, or trypsin 3-specific antibodies. Gain settings for the pulldowns were enhanced in order to observe faint bands (right panel).

For comparison, we also tested our previous NE probe, Cy5-V-DPP, in distal colon lysates and labeling of the 25-kDa species was barely distinguishable from the background (Fig. 5G). Thus, PK105b is clearly superior to Cy5-V-DPP for its ability to detect NE activity in tissue lysates. Both probes exhibit binding to several species in the 50–75-kDa range (Fig. 5A), and future proteomics assays will be required to determine their identity. Furthermore, we investigated secreted proteases found in the lumen of the colon (either luminal flush or in fecal pellets) with PK105b (Fig. 6A). In both samples, we observed two labeled proteases at 25 kDa. Immunoprecipitation confirmed low levels of NE in these samples, with pancreatic elastase and trypsin 3 being the predominant species (Fig. 6C). Nonetheless, NE activity could be clearly delineated by PK105b in lysates from colon tissues.

### Application of PK105b to measure NE activation in mucosal biopsies from IBD patients

**Figure 7:**
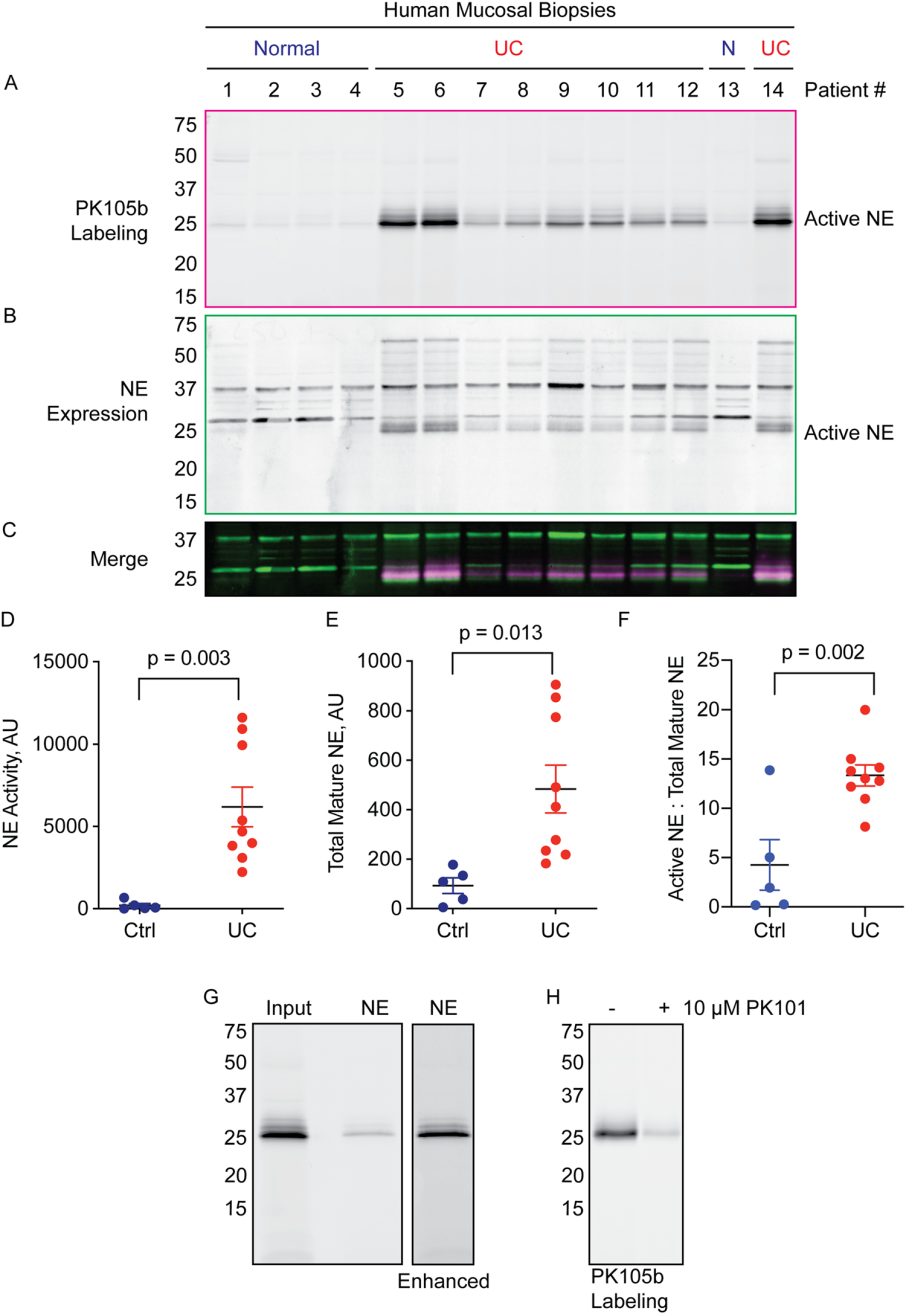
Application of PK105b to measure NE activation in mucosal biopsies from patients with IBD. (**A**) Mucosal biopsies from healthy controls (n = 5) or patients with active UC (n = 9) were labeled *ex vivo* with 1 µM PK105b. Probe binding was assessed by in-gel fluorescence. (**B**) Samples in (**A**) were immunoblotted for total NE expression. (**C**) Merged image of PK105b labeling (active NE; magenta) and immunoblot (total NE expression; green). (**D**,**E**) Densitometry of active NE in (**A**) and total mature NE in (**B**), respectively. (**F**) Ratio of active to total mature NE in the biopsy lysates. Error bars are shown as mean ± SEM. (**G**) Immunoprecipitation of PK105b-labeled UC biopsy lysates with an NE-specific antibody. Gain settings for pulldown were enhanced in order to view faint bands (right panel). (**H**) Competition of PK105b labeling in inflamed colon lysates with PK101.

To translate our findings in mouse colitis to human disease, we examined PK105b labeling in mucosal biopsies from patients (Table 1). As in mice, we observed a significant increase in labeling in samples from patients with active UC compared to healthy individuals brought in for routine colonoscopy screening (Fig. 7A,D). In contrast to mice, where we observed a single 25-kDa species at labeled by PK105b, three species were labeled in human mucosal lysates, with the smallest form having the most activity. The banding pattern resembled that which was observed with recombinant human NE (Fig. 2A and in refs^24,25^). We confirmed these bands to be NE by immunoprecipitation with an NE-specific antibody (Fig. 7G), and they could be competed with PK101 (Fig. 7H).

**Table 1:** Profiles of patients from which mucosal biopsies were collected.

Pt #	Symptoms	Medication	Pathology	Endoscopy
1	none	none	normal tissue	none
2	none	none	normal tissue	none
3	none	none	normal tissue	none
4	none	none	normal tissue	none
5	flare-up, 15–20 bm/d	Steroids, biologic	chronic inflammation, severe activity	pancolitis, Mayo 3 distal, 2 proximal
6	flare-up, 10 bm/d	none	chronic inflammation, moderate to severe activity	pancolitis, Mayo 2
7	chronic active, 4 bm/d	none	chronic inflammation, mild activity	proctitis, Mayo 1
8	chronic active, 2–3 bm/d	5-ASA	chronic inflammation, marked activity	pancolitis, Mayo 3 distal, 2 proximal
9	new onset, 6–8 bm/d	none	chronic inflammation, moderate activity	pancolitis, Mayo 2
10	chronic active, 12 bm/d*	Steroid enema, 5-ASA	chronic inflammation, mild activity	proctitis, Mayo 2
11	flare-up, 6–8 bm/d	Imuran	acute, chronic inflammation, deep ulcers	ileocolitis, deep ulcers
12	flare-up, 4–5 bm/d	5-ASA	chronic inflammation, severe activity	colitis
13	flare-up, pain, +2 bm/d	5-ASA	normal tissue; chronic stricture with no active inflammation	ileal stricture, blind biopsies
14	flare-up, 2–5 bm/day	none	chronic inflammation, moderate activity	pancolitis, Mayo 1–2

5-ASA = 5 aminosalicylic acid; *mucous, but infrequent stool; **suspected flare up initially but with further imaging dx. with chronic stricture with no active inflammation; bm = bowel movement, most were bloody.

Furthermore, when the same samples were immunoblotted for total NE expression, we observed NE bands in the healthy tissue at 37 and 25 kDa (Fig. 7B). UC tissues, however, displayed an additional doublet that was smaller than the 25-kDa species. The most active species, as indicated by PK105b labeling, corresponded to these smaller species (Fig. 7C,G). Thus, as we observed in mouse colitis, NE undergoes trimming during human UC that permits its activation and binding to PK105b.

Patient #13 was originally diagnosed with UC and we initially considered this biopsy to be an outlier given the low levels of NE activation. Upon follow-up studies, however, this diagnosis was re-evaluated and the patient was found to have an ileal stricture. The biopsies analyzed in this study were taken from normal tissue regions. These data therefore highlight the potential utility of using NE activity as a diagnostic marker for ulcerative colitis.

## Discussion

While PK105 was selective for NE in purified neutrophils, its ability to label NE in tissue lysates, which contain many cell types and proteases, had not been established. We tested PK105b, a close analogue of PK105, in bone marrow, pancreas, inflamed lungs, oral cancer, and human and mouse colitis tissues and fecal samples. Due to the covalent nature of PK105b, we could verify that NE was clearly targeted by this probe. We also observed that PK105b exhibited cross-reactivity with other serine proteases such as pancreatic elastase, proteinase-3, trypsin 3 and other unknown proteases. This cross-reactivity was dependent on the tissues examined, with greater selectivity observed in purified lung neutrophils, lung tissue, and human colon biopsies and more cross-reactivity in pancreas, fecal samples, and cancer tissue. We confirmed that all PK105b binding was mediated by the DPP warhead in combination with the specificity region, and not due to non-specific binding via the Cy5 fluorophore, as all labeling could be blocked by pre-treatment with PK101, a non-fluorescent analogue of PK105.

While PK105b clearly has limitations, it is advantageous over traditional substrate probes such as AAPV-p-nitroanilide and BODIPY-FL-elastin, as these tools do not bind their targets covalently and the extent of their cross-reactivity in various contexts cannot readily be determined. Additionally, PK105b was advantageous over our previous diphenylphosphonate probe, Cy5-V-DPP^13^. In competition assays with the fluorogenic substrate, AAPV-AMC, PK105b reacted with rNE much more rapidly and potently than Cy5-V-DPP. This is most likely mediated by the improved non-natural specificity region in PK105b, which may bind more tightly within the NE active site. PK105b may also have enhanced sensitivity in tissue lysates due to increased stability, as its non-natural peptide sequence may be more resistant to degradation by other proteases than valine. PK105b is therefore one of the most effective tools available to study NE activation.

We applied PK105b to investigate NE activation during mouse colitis. In inflamed colons, we observed labeling of active NE, which was significantly increased compared to control tissue. Probe-labeled NE corresponded to the most mature species of NE detected by immunoblotting, which was not present in healthy tissue. We corroborated these findings in human colitis using mucosal biopsies from patients with UC, unequivocally demonstrating for the first time that NE is activated in IBD. Thus, PK105b is well suited for detection of both mouse and human NE during IBD and will be a valuable resource for future investigation of NE function. Furthermore, it may have utility as a diagnostic agent for IBD and for validating target engagement and efficacy of neutrophil elastase inhibitors in preclinical development as therapeutic agents.

## Experimental Procedures

### Probe synthesis and characterization

Refer to Supplemental Materials for information on the synthesis of PK105b. Cy5-V-DPP^13^ and PK101^14^ were synthesized in house as described previously.

### Recombinant protease labeling

Recombinant proteases (500 ng) were diluted in 20 µl of phosphate-buffered saline (PBS): human neutrophil elastase (Elastin Products Company), porcine pancreatic trypsin type II-S (beta trypsin; Sigma), and human proteinase-3 (Sigma). PK105b or Cy5-V-DPP (0, 0.1, 0.5 or 1 µM) was added from a 100x DMSO stock, and reaction was carried out at 37 °C for 30 minutes. Proteins were solubilized in 4x sample buffer (40% glycerol, 200 mM Tris-Cl [pH 6.8], 8% SDS, 0.04% bromophenol blue, 5% beta-mercaptoethanol), boiled and resolved on a 15% SDS-PAGE gel under reducing conditions. Probe labeling was detected by scanning the gel for Cy5 fluorescence on a Typhoon 5 flatbed laser scanner (GE Healthcare). Detailed protocols for ABP application are available in ref.^26^.

### Fluorogenic substrate assay

Recombinant neutrophil elastase (20 nM) was diluted in PBS, and MeOSuc-Ala-Ala-Pro-Val-AMC (AAPV-AMC; 20 µM; Bachem) was diluted in PBS containing PK105b (0.2 µM), Cy5-V-DPP (2 or 20 µM), sivelestat (2 µM; Sigma) or 2% DMSO (vehicle control). The two solutions were mixed 1:1 in an opaque 96-well plate (100 µl final volume), and probe-dependent fluorescence (355 nm excitation/460 nm emission) was immediately measured at 30 s intervals over the course of 1 h on a FLUOstar Omega plate reader (BMG Labtech).

### Animal ethics

All animal experiments were conducted in accordance with the guidelines for the use of laboratory animals in research and approved protocols. Experiments involving healthy mice and colitis experiments were approved by the Monash University Animal Ethics Committee. Oral cancer experiments were approved by the Committee on Animal Research at New York University. Legionella experiments were approved by the University of Melbourne Animal Ethics Committee.

### *Ex vivo* tissue labeling

Bone marrow was obtained by flushing tibias and femurs from healthy C57BL/6J mice with PBS. Cells were washed and resuspended in PBS prior to sonication on ice. Tissues were lysed by sonication on ice in PBS (10 µl/mg tissue), and supernatants were cleared by centrifugation at 21,000 g for 10 min at 4 °C. Total protein (60 µg, as measured by BCA assay, Pierce) was aliquoted in a total volume of 20 µl PBS, and probe labeling and SDS-PAGE was carried out as above. Where indicated, PK101 (10 µM) was added for 10 minutes prior to the addition of PK105b.

### Western blotting

Fluorescent gels were transferred to nitrocellulose membranes and blotted using the Turbo Blot system (BioRad). Membranes were blocked using Li-Cor Odyssey blocking buffer diluted by 50% with PBS containing 0.05% Tween 20. Sheep anti-mouse neutrophil elastase/ELA2 (1:1000; R&D AF4517) was incubated overnight at 4 °C. Secondary antibody (goat-IR800, 1:5000; LiCor) was incubated for one hour at room temperature. Binding was detected by scanning with the IRLong filter on the Typhoon 5.

### Immunoprecipitation

PK105b-labeled lysates (boiled in sample buffer) were divided into input and immunoprecipitation (IP) samples (100 µg each). The IP samples were diluted in 500 µl IP buffer (PBS [pH 7.4], 1 mM EDTA, 0.5% NP-40) along with 10 µl antibody: Sheep anti-neutrophil elastase/ELA2 (R&D AF4517); rabbit anti-PRSS3 (Abcam ab105123); rabbit anti-pancreatic elastase (Abcam ab21593). Protein A/G beads (40 µl slurry; Santa Cruz) were washed with IP buffer and then added to the sample. Tubes were rocked overnight at 4 °C. Beads were washed four times with IP buffer and once with 0.9% sodium chloride. After the last wash, all buffer was removed and beads were boiled in 2x sample buffer (20 µl). Supernatants were then analyzed, alongside the input sample, by fluorescent SDS-PAGE as above.

### *Legionella pneumophila* infection model

C57BL/6 mice were infected by intranasal inoculation with 2.5 × 10^6^ *L. pneumophila* 130b ΔflaA in 50 μL of PBS. Three days after infection, lungs were collected, minced and digested in 4 mL RPMI-1640 (Gibco) containing 3% FCS, 1 mg/mL DNaseI (Sigma-Aldrich) and 1 mg/mL Collagenase III (Worthington). Cells were collected by filtration through a 70-μM filter, centrifuged and red blood cells lysed by resuspension in buffer containing 150 mM ammonium chloride and 50 mM Tris-HCl (pH 7.5) for 5 min. After washing with PBS containing 0.1% BSA and 2 mM EDTA, cells were stained with anti-Ly6G-FITC (1A8, BD Pharmingen), anti-CD11c-PE (N418, eBioscience), anti-Siglec-F-BV421 (E50-2440, BD Horizon), anti-CD64-Alexa 647 (X54-5/7.1, BD Pharmingen) and anti-FcγII/III (2.4G2, WEHI monoclonal facility) for 30 min at 4 °C. Cells were washed and resuspended in FACS buffer with 0.25 μg/mL 7-AAD. Neutrophils were isolated using a Beckman Coulter MoFlo Astrios cell sorter into FCS and pellets of 10^6^ cells snap frozen at −80 °C. Purities were assessed post-sorting using the same gating strategy. Purified neutrophils were lysed on ice in PBS with 0.1% Triton X-100, and supernatants were cleared by centrifugation. Alternatively, lung tissues from the same mice were snap frozen at the time of harvest and processed as above.

### Oral cancer model

Female BALB/c nude mice (6–8 weeks old, Charles River Laboratories) were injected in the left lateral tongue under anesthesia (3 × 10^5^ HSC-3 human oral squamous cell carcinoma cells suspended in 50 µl vehicle [1:2 mixture of DMEM and Matrigel; Becton Dickinson], or vehicle alone). After two weeks, the resulting xenografted tumors and vehicle-injected tongues were excised, snap frozen, and analyzed as above.

### Colitis model

Mice were purchased from the Monash University in-house colony. Colitis was induced in 10-week old male C57BL/6J mice by intracolonic infusion of picrylsulfonic acid solution (2,4,6-Trinitrobenzenesulfonic acid solution, TNBS; Sigma; 2.5 mg dissolved in 50% ethanol). Body weight and symptoms were recorded daily, and mice were humanely killed after three days. Upon colon extraction, luminal fluids were collected by flushing colons with PBS. Solids were removed by centrifugation and supernatant was concentrated using a 3-kDa cut-off centrifugal filter (Amgen). Pieces of proximal and distal colon were frozen for protease analysis or fixed in 4% paraformaldehyde overnight, paraffin embedded, sectioned, and stained with haematoxylin and eosin.

### Human mucosal biopsies

Human mucosal biopsies were obtained from individuals during colonoscopy procedures at Hotel Dieu Hospital in Kingston, Ontario, Canada. Informed written and verbal consent was obtained prior to enrolment and all protocols were approved by and carried out in accordance with the guidelines and regulations of the Queen’s University Human Ethics Committee. Patients were well-characterized individuals with active UC or healthy individuals undergoing routine colonoscopy for cancer screening (Table 1). For UC patients, biopsies were obtained from sites of active inflammation. Fresh biopsies were washed in PBS and then snap frozen for protease analysis as above.

### Statistical analysis

All experiments were performed with at least three biological replicates. Data are reported as means ± SEM. Statistical significance was determined by comparing two groups using a Student’s t test, and p values of less than 0.05 were considered significant.

## Supplementary information

Supplementary Information

## Data Availability

All data generated or analyzed during this study are included in this published article (and its Supplementary Information files).
