# Supplementary material for: Application of a chemical probe to detect neutrophil elastase activation during inflammatory bowel disease
Source: Sci Rep. 2019 Sep 16;9:13295. doi: 10.1038/s41598-019-49840-4 (PMC6746801; doi:10.1038/s41598-019-49840-4)
Supplement: Supplementary file 1 — Supplementary Information [file 41598_2019_49840_MOESM1_ESM.pdf]

Supplementary Information for:

Application of a chemical probe to detect neutrophil elastase activation during inflammatory bowel disease

Bethany M. Anderson[1,2], Daniel P. Poole[2], Luigi Aurelio[3], Garrett Z. Ng[1], Markus Fleischmann[1,4], Paulina Kasperkiewicz[5], Celine Morissette[6], Marcin Drag[5], Ian R. van Driel[1], Brian L. Schmidt[7], Stephen J. Vanner[6], Nigel W. Bunnett[2,8,9] & Laura E. Edgington-Mitchell[1,2,7*]

Supplemental Methods: Synthesis and characterization of PK105b

Fmoc amino acids were purchased from Chem-Impex and Novabiochem, coupling reagents were purchased from GL Biochem, and solvents and other reagents were purchased from Merck and used without further purification. Resins were purchased from Chem-Impex and Cy5-acid was purchased from Lumiprobe.

Reverse Phase-HPLC purification of crude peptides was performed on an Agilent 1200 quaternary pump system, photodiode array detector (214 nm), employing a Phenomenex Axia column (Luna C8(2), 50 x 21.3 mm ID) eluting with a gradient of 5-100% of 0.1% TFA/acetonitrile in 0.1% aqueous TFA, over 60 minutes at a flow rate of 10 mL/min. Appropriate fractions collected were analyzed by LC-MS on an Agilent 1260SQ system, incorporating a photodiode array detector (214 nm) coupled directly to an API-ES quadrupole mass analyser. The combined fractions were freeze-dried for two days to give the purified peptides as TFA salts and their purity was >90% as estimated by reversed-phase HPLC carried out employing a Poroshell 120 EC-C18 3.0 X 50mm 2.7-Micron eluting with a gradient of 5-100% acetonitrile in 0.1% aqueous formic acid, over 3.8 min and maintained to 100% acetonitrile until 5 min at a flow rate of 0.5 mL/min, detection was at 214 nm. The compounds were confirmed as having the correct molecular weight by API-ES MS analysis. Mass spectra were acquired in negative ion mode with a scan range of 200-2000 m/z.

sulfoCy5-Nle(OBzl)-Met(O)$_2$-Oic-OH

Synthesis of the protected linear peptide (Cy5-Nle(OBzl)-Met(O)$_2$-Oic-OH) was carried out using manual peptide synthesis with standard Fmoc solid phase peptide chemistry. Synthesis was undertaken using Chlorotrityl chloride resin (loading 1.0 mmol/g from Chem-Impex) on a 0.2 mmol scale (0.3 g of resin). Coupling of the first amino acid was performed with Fmoc-Oic-OH (1.2 mol eq relative to resin loading) in dichloromethane (DCM) activated with 3 mol eq of diisopropylethylamine (DIPEA). This was carried out overnight at room temperature. The resin was then washed with DMF (3 × 5 mL × 2 min each and then DCM 2 × 5 mL× 2 min each) and then exposed to the deprotection solution 20% piperidine in DMF (3 × 5 mL × 5 min each) and after the third deprotection step a positive bromophenol blue test resulted. Coupling of subsequent Fmoc-amino acids were performed using the 1.5 mol eq. (relative to resin

loading) of Fmoc amino acid, PyBOP (1H-Benzotriazol-1-yloxy)(tri-1-pyrrolidinyl)phosphonium hexafluorophosphate in DMF (5 mL/g of resin) with activation in situ, using 3 mol equiv of DIPEA. This was carried out for 1 h at room temperature (RT). At this stage the TNBS test was used to monitor peptide coupling providing a negative result. The resin was then washed with DMF (3 × 5 mL × 2 min each and then DCM 2 × 5 mL× 2 min each). The resin was then exposed to the deprotection solution 20% piperidine in DMF (3 × 5 mL × 5 min each) and after the third deprotection step a positive TNBS test resulted. The resin was washed with DMF (3 × 5 mL × 2 min each and then DCM 2 × 5 mL× 2 min each) and the coupling process continued with the next Fmoc amino acid until the sequence was completed. The final amino acid on the peptide resin was Fmoc deprotected with 20% piperidine in DMF (3 × 5 mL × 5 min each) and then thoroughly washed with DMF then DCM. A portion of the resin (30 mg, 0.03 mmol) was suspended in 4:1 DMF:DMSO and sulfoCy5 acid (30 mg, 0.046 mmol) was added to the mixture followed by PyBOP (0.1 mmol) and finally DIPEA (0.6 mmol). The mixture was left for 24h with intermittent agitation and then thoroughly washed with DMSO (until a colorless filtrate was obtained), followed by DMF, DCM, MeOH and finally Ether. The dried resin was taken up in 5 mL of HFIP (hexafluoroisopropanol):DCM:TIPS (v:v:v, 30:69:1) and left to stand for 2h. The filtrate was filtered from the resin and the resin washed with HFIP until colourless. The combined filtrate and washings were concentrated to a residue (10.1 mg) and then purified by RP-HPLC providing 3mg of the intermediate sulfoCy5-Nle(OBzl)-Met(O)$_2$-Oic-OH as a blue powder. The compound was confirmed as having the correct molecular weight by API-ES analysis: *m/z* calculated; $C_{60}H_{79}N_5O_{14}S_3$ $[M - H]^-$ 1189.5, $[M - 2H]^{2-}$ 593.7; observed: $[M - H]^-$ 1189.0, $[M - 2H]^{2-}$ 593.6.

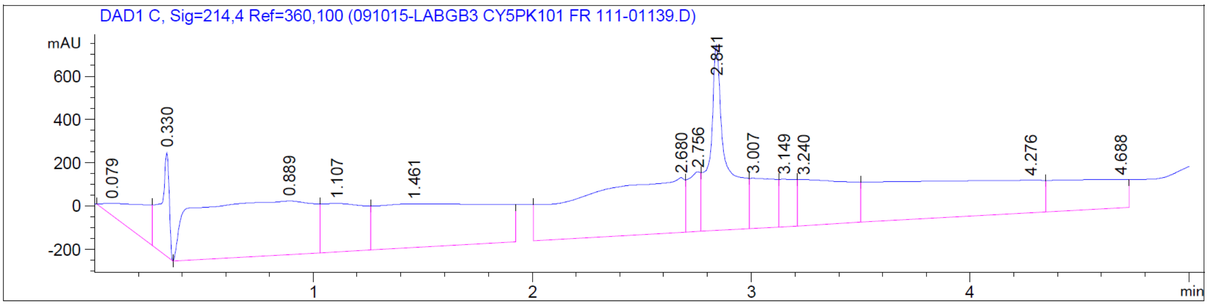

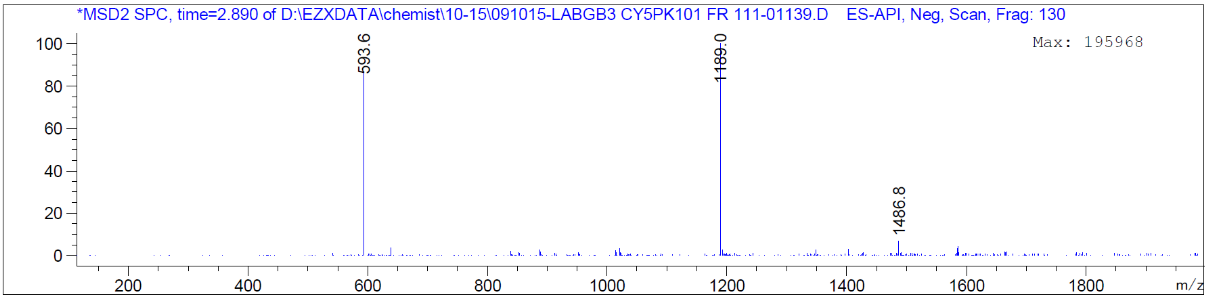

PK105b

Cy5-Nle(OBzl)-Met(O)$_2$-Oic-OH (1mg) was taken up in dry DMSO (50 µL) in an Eppendorf tube (1.5 mL) and to this mixture was added PyBOP (2 mol eq), Abu^P(OPh)$_2$.HBr (1.2 mol eq) followed by DIPEA (6 mol eq). The mixture was agitated for 24h and then diluted in ACN (6 mL) and purified by RP-HPLC providing 0.7mg of the final compound PK105b as a blue powder. The compound was confirmed as having the correct molecular weight by API-ES analysis: *m/z* calculated; $C_{75}H_{95}N_6O_{16}PS_3$ $[M - H]^-$ 1462.8, $[M - 2H]^{2-}$ 730.3; observed: $[M - H]^-$ 1462.8, $[M - 2H]^{2-}$ 730.2.

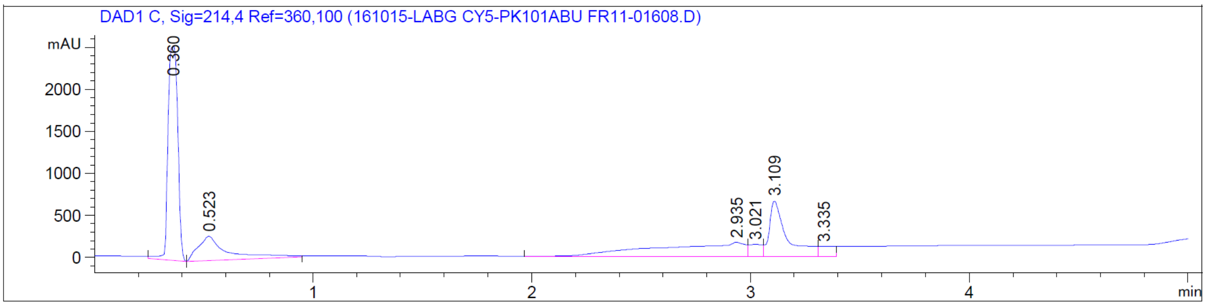

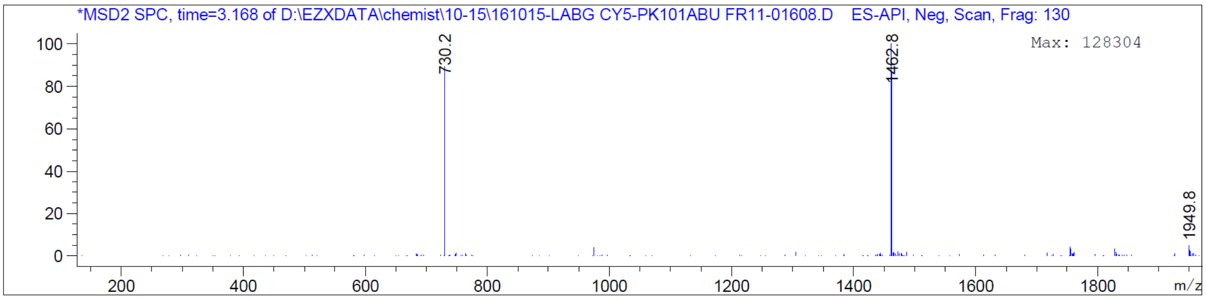

SUPPLEMENTAL FIGURE 1

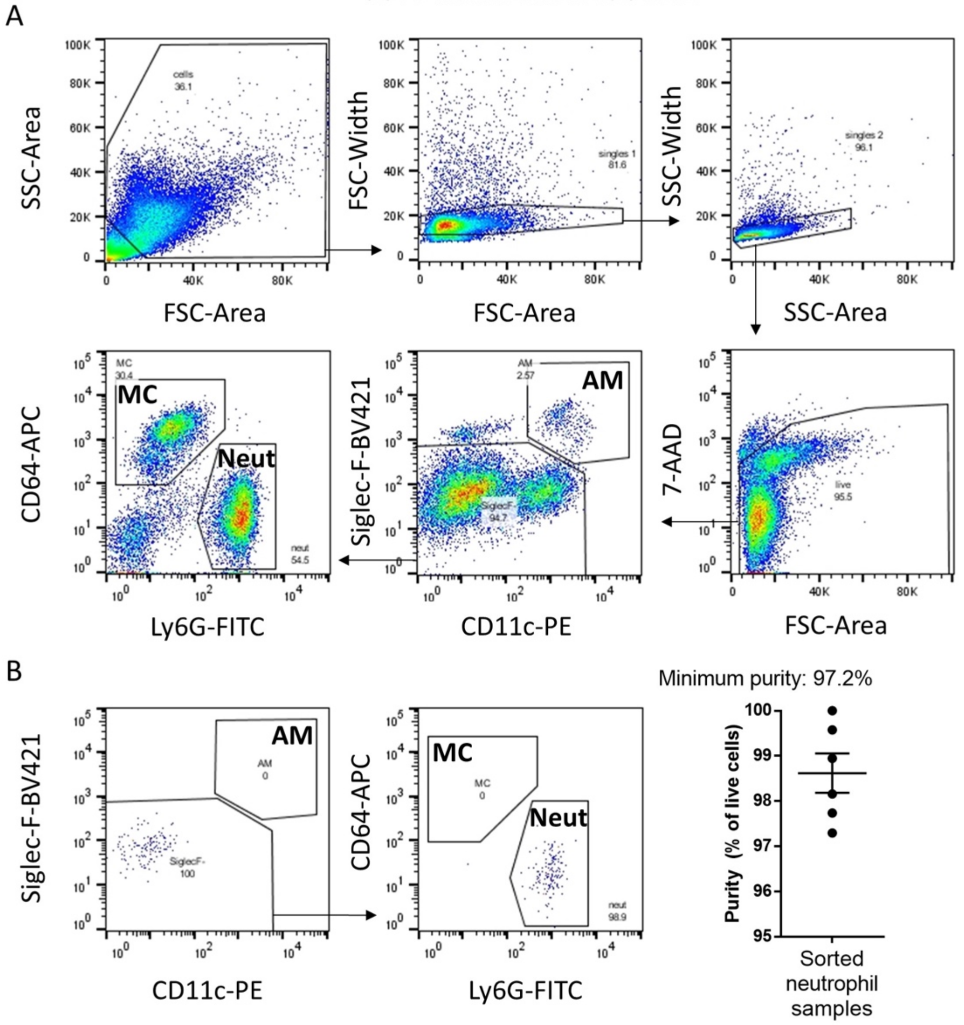

Figure S1. Neutrophil sorting from *L. pneumophila*-infected lung tissues. (A) Single-cell suspensions were prepared from infected lung tissues and stained with a panel of antibodies tailored to distinguish neutrophils from alveolar macrophages (AM) and monocytic-like cells (MC). Siglec-F$^-$/CD11c$^-$/CD64$^-$/Ly6G$^+$ cells were sorted. (B) A subset of sorted cells from each sample was re-analyzed, revealing that the neutrophil populations were >97% pure. Error bars are shown as mean ± SEM. n= 6.

SUPPLEMENTAL FIGURE 2

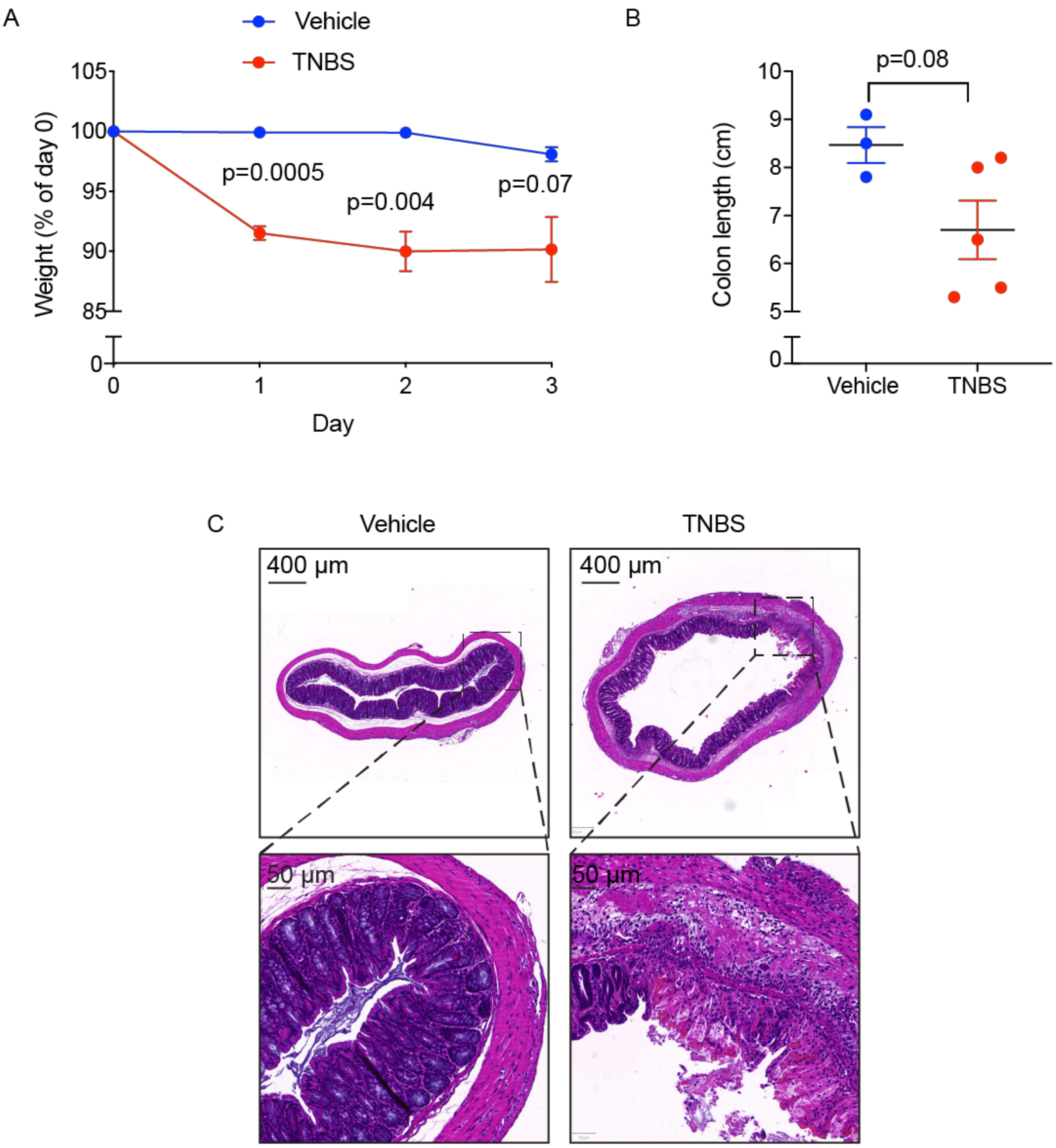

Figure S2. Verification of colitis symptoms in response to TNBS induction. (A) Body weight, normalized to day 0, of healthy mice or those with TNBS-induced colitis. (B) Colon length at the endpoint of the colitis experiment (day 3). (C) H&E staining of colons with and without TNBS-induced colitis. Error bars are shown as mean ± SEM. n= 3-5.
